# A preliminary study of primary retroperitoneal sarcoma at a tertiary University Hospital in Bangkok, Thailand: a retrospective observational study

**DOI:** 10.2478/abm-2024-0031

**Published:** 2024-10-31

**Authors:** Suvit Sriussadaporn, Sukanya Sriussadaporn, Rattaplee Pak-Art, Kritaya Kritayakirana, Supparerk Prichayudh, Pasurachate Samorn, Natawat Narueponjirakul, Punthita Aimsupanimitr, Apinan Uthaipaisanwong

**Affiliations:** Department of Surgery, Faculty of Medicine, Chulalongkorn University, Bangkok 10330, Thailand

**Keywords:** complete resection, multivisceral resection, preoperative radiation, primary retroperitoneal sarcoma, sarcoma recurrence

## Abstract

**Background:**

Retroperitoneal sarcoma (RPS) is rare and difficult to treat with a high recurrent rate. Very little data regarding primary RPS exists in Thailand.

**Objectives:**

To study the outcome of treatment of primary RPS at a tertiary University Hospital in Bangkok, Thailand.

**Methods:**

All patients who had RPS undergoing the first surgical resection at King Chulalongkorn Memorial Hospital from June 2003 to December 2019 were retrospectively enrolled in the study. Perioperative management, results of treatment, postoperative complications, and outcome were analyzed.

**Results:**

Thirty-eight patients entered the study. Large abdominal mass was the most common presentation (90%). Liposarcoma was the most common histology (58%). Twenty patients (53%) had preoperative core needle biopsy and 21 (55%) underwent preoperative radiotherapy (RT). The tumor size ranged from 3 cm to 48 cm (median 22 cm). Five patients (13%) had total mass removal only while 33 (87%) had complete gross resection with ≥1 visceral organ resection. Surgical margins classified as R0, R1, and R2 were 61%, 34%, and 5%, respectively. Five patients (16%) had postoperative complications. There was no 30-day postoperative mortality. The local recurrence rate was 34%. Survival analysis revealed a 5-year overall survival rate of 37% and 5-year disease-free survival rate of 29%. The 5-year and 10-year recurrent rates were 71% and 95%, respectively. Multivariate analysis showed that preoperative radiation was the only factor reducing recurrence (19% vs. 53%, OR: 0.21, *P* = 0.011).

**Conclusion:**

The preliminary study of outcome of the treatment of primary RPS at our institution showed a fair prognosis of this rare malignancy despite our aggressive surgical approaches. Preoperative radiation may help reduce recurrence in selected primary RPS patients.

Sarcoma is a group of malignant tumors arising from connective tissue which is derived from the embryonic mesoderm. Retroperitoneal sarcoma (RPS) is a malignant soft tissue tumor located in the retroperitoneal area. It is a rare malignancy with approximately 0.5–1 new cases per 100,000 population per year [1]. Treatment is usually difficult owing to its large size at first presentation, which puts immense pressure on the surrounding major vascular and visceral structures. Compared to other solid malignancy, RPS is relatively less well-known in terms of proper management and a higher rate of recurrence leading to poor outcome [2, 3]. During the last 3 decades, several advances have been made in the management of RPS resulting in new concepts of treatment from a tumor with a low rate of resection to a higher rate of successful complete resection [4, 5]. In Thailand, we reported our surgical experience of both primary and recurrent RPS in 18 patients in 2014 [6]. This article is, to the best of our knowledge, the largest case series of patients suffering from RPS in Thailand. However, while the natural history and outcome of treatment of RPS have been intensely studied and firmly established in North America and Europe during the past 2 decades [7,8,9,10], very little data on this rare malignancy exists in Thailand. Sporadically, some reports came from Asia and Australia [11,12,13,14].

King Chulalongkorn Memorial Hospital, which is a 1300-bed tertiary care medical center located in the heart of Bangkok, Thailand, has been dedicated to being a referral center for patients with RPS for >15 years. Patients who came to our surgical services were those who had never been treated before (primary RPS) and those who were sent to us with recurrent disease after previous surgical removal of primary tumors elsewhere. This study aimed to examine patients with primary RPS who underwent the first surgical treatment at our institution. Details of the study included clinical presentations; preoperative, operative, and postoperative management; postoperative complications; histology of the tumors; results of treatment; recurrence rate; and survival analysis.

## Methods

This is a retrospective observational cohort study of patients who had primary RPS and underwent the first surgical treatment at King Chulalongkorn Memorial Hospital from June 2003 to December 2019. The study was approved by the Ethics Committee of the Institutional Review Board (IRB) of the Faculty of Medicine, Chulalongkorn University (IRB No. 497/64). All patients had computed tomography (CT) scan of the abdomen which revealed retroperitoneal tumors. Before 2009, patients with suspected RPS (6) received surgical resection with neither preoperative tissue diagnosis nor preoperative radiotherapy (RT). Preoperative core needle biopsy has become our standard approach for tissue diagnosis of RPS since 2009. Core biopsy (under CT scan or ultrasound guided) was performed by interventional radiologists to confirm definite preoperative diagnosis of RPS. Preoperative RT has been introduced to our institution for preoperative management (total dose of 50–60 Gy over 6-week period) since 2011. Preoperative RT was not considered in patients who had an extremely large retroperitoneal mass with severe compressive symptoms at the first presentation to prevent further delay of surgical treatment. Some patients with preoperative CT scan compatible with a resectable well-differentiated liposarcoma were also exempted from preoperative core biopsy and preoperative RT. In patients who underwent preoperative RT, operations were scheduled at 6–8 weeks after completion of a 6-week period of RT. Only patients who underwent curative-intent resection were included in this study. Exclusion criteria were patients with sarcomatosis, distant metastasis at first presentation, and concomitant malignancy of other organs.

All operations were carried out by one surgical team using uniform operative techniques. Surgery aimed to totally remove the tumor (en-bloc resection) together with the surrounding organs (multivisceral resection) to obtain adequate clearance of the surgical margins from any residual tumor cells. Such an approach resulted in total mass removal with multivisceral resection or compartmental resection [6, 15]. Details of the operation have been described in previous reports [6, 15]. Postoperatively, patients were monitored in the surgical intensive care unit for 1–2 days. Reoperation was immediately performed when postoperative intraabdominal hemorrhage was diagnosed or strongly suspected. Massive hemorrhage was a serious complication following surgical treatment of RPS owing to the large raw surface area created during the operation. Furthermore, the intraabdominal cavity could contain several liters of blood without abdominal distension because of the already expanded intraabdominal cavity from huge retroperitoneal tumor.

Surgical specimens were sent to the Pathology Department for definite pathological diagnosis and determination of adequacy of the tumor margins. The presence or absence of residual tumors was classified as R0, R1, and R2 resections (R0: no macroscopic and microscopic tumors, R1: positive microscopic margins only, and R2: gross tumor left in the operative field). The histologic grading was made according to the French grading and the National Cancer Institute grading (FNCLCC Grading System) [16]. The French grading system is based on 3 parameters, i.e., tumor differentiation (score 1, 2, and 3), mitotic index (score 1, 2, and 3), and tumor necrosis (score 0, 1, and 2). Histologic grading is performed by summing the scores obtained from these 3 parameters. The results are grade 1 (total score 2 or 3), grade 2 (total score 4 or 5), and grade 3 (total score 6–8). Grade 1 represented low-grade tumor and grades 2 and 3 represented high-grade tumor with poorer prognosis.

A follow-up visit was scheduled every 4–6 months during the first 5 years postoperatively, then yearly afterward for life. CT scan was used as an important imaging tool to detect recurrence. Early detection of recurrence was the goal of postoperative follow-up since further appropriate management could be initiated in a timely manner.

Statistical analysis was performed using the Window SPSS program (IBM Corp., Armonk, New York, 2013). with the statistical significance set at *P* < 0.05. Comparison of categorical variables was performed with a chi-square test (with *P-*values reported by using twice one-tailed exact probability). The local recurrence-free survival and overall survival in each tumor grade were compared using Kaplan–Meier analysis and the log rank test.

## Results

During the 16.5-year study period, 38 patients with primary RPS underwent the first operation at our Surgical Unit, King Chulalongkorn Memorial Hospital, Bangkok, Thailand. All RPS patients were diagnosed from abdominal CT scan. Details of patient characteristics and demographic data are shown in **Table 1**. The sarcoma histology, surgical margins, and tumor grading are shown in **Table 2**. Complete resection (R0/R1) was the aim of surgical treatment, this resulted in complete tumor resection with >1 visceral organ resection in 33 patients (87%). Five patients (13%) had total mass removal only. The 3 most common organs resected en-bloc with the tumor were kidney 22 (58%), colon 18 (43%), and uterus with both fallopian tubes 8 (21%) (**Table 3**). Postoperative complications occurred in 5 patients (16%). Details of complications are shown in **Table 4**. There was no 30-day postoperative mortality.

**Table 1.** Patient characteristics and demographic data

Study period	June 2003–December 2019
Number of patients (n)	38
Male (n)	11 (29%)
Female (n)	27 (71%)
Age (years)	26–80 (median 54)
Duration of symptoms (months)	1–24 (median 4)
Tumor size (cm)	3–48 (median 22)

**Presentations** (some patients had >1 presentation)	**No (%)**

Abdominal mass	34 (90)
Abdominal pain, discomfort	8 (21)
Anorexia	1 (3)
Incidental finding during appendectomy	1 (3)
Incidental finding during gynecologic procedures	2 (5)
Asymptomatic	1 (3)
Preoperative core needle biopsy	20 (53)
Previous operation with biopsy	7 (18)
Preoperative RT	21 (55)
Postoperative RT	16 (42)
Location of tumors	
Right	17 (45)
Left	13 (34)
Pelvis	8 (21)

RT, radiotherapy.

**Table 2.** Sarcoma histology, surgical margins, and tumor grading

**Histologic diagnosis**	**No (%)**	
Liposarcoma		
Well differentiated	5	(58)
Dedifferentiated	9	
Myxoid	1	
Pleomorphic	1	
Mixed type	6	
Leiomyosarcoma	4 (11)	
Malignant peripheral nerve sheath tumor	1 (3)	
Malignant fibrous histiocytoma (undifferentiated pleomorphic sarcoma)	1 (3)	
Mixofibrosarcoma	1 (3)	
Rhabdomyosarcoma	1 (3)	
Spindle cell sarcoma	8 (21)	
Surgical margins		
R0	23 (61)	
R1	13 (34)	
R2	2 (5)	
Tumor grading		
Grade 1	15 (40)	
Grade 2	7 (18)	
Grade 3	16 (42)	

**Table 3.** Number of organs resected in 33 patients (87%)

**Organs**	**No (%)**
Kidney	22 (58)
Colon	18 (43)
Uterus and both fallopian tubes	8 (21)
Adrenal gland	4 (11)
Spleen	4 (11)
Distal pancreas	3 (8)
Inferior vena cava	2 (5)
Diaphragm (partial)	2 (5)
Wedge resection of duodenum	1 (3)
Segmental resection of ureter with end-to-end anastomosis	1 (3)
Paraaortic lymphnodes	1 (3)
Left uterine tube	1 (3)

RT, radiotherapy.

**Table 4.** Postoperative complications

**Complications**	**No of pt.**	**Management**
Intraabdominal bleeding	3	Reoperation with bleeding control
Wound dehiscence	1	Reoperation with re-suturing abdominal wound
Prolonged ileus	1	Conservative treatment

Eight patients (21%) received doxorubicin-based chemotherapy postoperatively; all of them had high-grade tumors with unfavorable histologic subtypes, while 4 had R2 resection.

**Figure 1.**
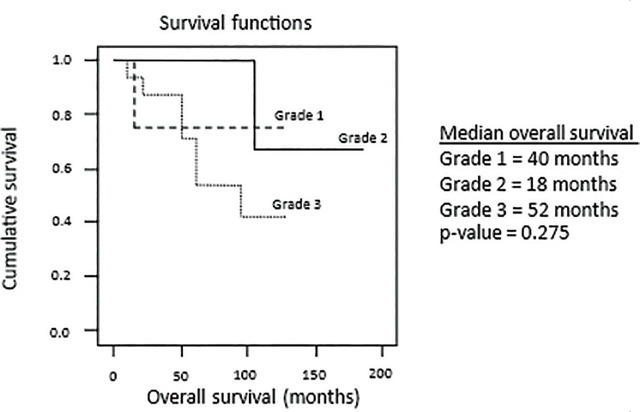
Kaplan–Meier plots of overall survival.

**Figure 2.**
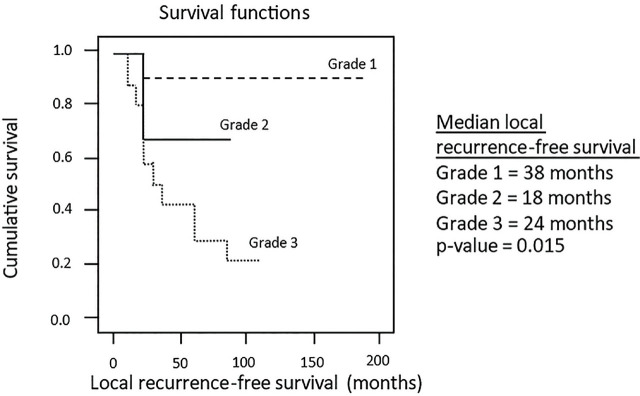
Kaplan–Meier plots of local recurrence-free survival.

At the end of the study (December 2019), 22 patients (58%) were alive without recurrence. Seven patients (18%) were alive with recurrence. Nine patients (24%) had died, 6 (67%) died of recurrence, and 3 (33%) died of other diseases without recurrence (2 chronic renal disease and 1 gastric cancer). The local recurrent rate was 34% (13 out of 38 patients). The median follow-up time was 46.5 months (interquartile range [IQR] 17, 97). Survival analysis revealed the 5-year overall survival rate of 37% and the 5-year disease-free survival rate of 29%. The median overall survival for tumor grade 1, grade 2, and grade 3 was 40, 18, and 52 months, respectively (NS) (**Figure 1**). The median local recurrence-free survival for tumor grade 1, grade 2, and grade 3 was 38, 18, and 24 months, respectively (*P* = 0.015) (**Figure 2**). Median survival time was 46.5 months (IQR 17, 97). The 5-year and 10-year recurrence rates were 71% and 95%, respectively. In 15 patients with low-grade tumor (grade 1), only 1 (7%) had recurrence. Of the remaining 23 (61%) patients with high grade (grade 2 or 3), 12 (52%) had recurrence. In 22 patients with liposarcoma, 6 (27%) with favorable histologic subtypes of well-differentiated and myxoid had no recurrence. However, of the 16 patients (73%) with dedifferentiated, pleomorphic, and mixed type, 9 (56%) had recurrence.

Univariate analysis revealed that high-grade tumors (grades 2 and 3) were significantly associated with recurrence compared to low-grade tumors (grade 1, *P* = 0.008). In liposarcoma, the histologic subtypes of well-differentiated and myxoid had significantly less recurrence than the dedifferentiated, pleomorphic, and mixed type (*P* = 0.046) (**Table 5**). However, multivariate analysis showed that preoperative radiation was the only factor reducing recurrence (19% vs. 53%, OR: 0.21, *P* = 0.011) (**Table 6**). Among 13 patients with recurrence (11 local recurrence, 4 liver metastases, and 1 lung metastasis), 7 underwent surgical resection, 4 received chemotherapy, 3 received radiation, and 2 received palliative care. Six patients with recurrence died of the sarcoma (46%).

**Table 5.** Univariate analysis of recurrence

**Variables**	**No recurrence (N = 25)**	**Recurrence (N = 13)**	***P*-value**
Age (years)			
<50	10	6	0.981
>50	15	7	
Tumor grading			
Grade 1	14	1	0.008
Grade 2 and 3	11	12	
Surgical margins			
R0/R1	25	11	0.222
R2	0	2	
Tumor size (cm)			
<15	9	5	1
>15	16	8	
Preoperative RT			
Yes	17	4	0.064
No	8	9	
Adjuvant chemotherapy			
Yes	1	7	0.002
No	24	6	
Postoperative RT			
Yes	9	7	0.476
No	16	6	
>1 visceral organ resection			
Yes	20	12	0.643
No	5	1	
Histologic subtype of liposarcoma			
Well differentiated, myxoid	6	0	0.046
Dedifferentiated, pleomorphic, mixed type	7	9	

RT, radiotherapy.

**Table 6.** Multivariate analysis of recurrence

**Step**	**Variable**	**Adjusted OR (95% CI)**	***P*-value**
1	Preoperative RT	0.21 (0.25–0.89)	0.011

Stepwise regression: variables included in the model were age, tumor grading, surgical margin, tumor size, preoperative RT, postoperative RT, adjuvant chemotherapy, visceral organ resection, and histologic subtype.

RT, radiotherapy.

## Discussion

RPS is rare and difficult to treat resulting in poor outcome notoriously perceived before 1980 [17]. In 1981, Cody et al. [18] from Memorial Sloan Kettering Cancer Center in New York reported an improvement in complete resection rate from 21% to 56% compared to a previous study at the same institution with operative mortality decreased from 11% to 2%. Since then, the concept of complete resection has become an important armamentarium in the management of patients with RPS. However, despite complete resection, recurrent rates were still high: 72% at 5 years and up to 91% at 10 years [19]. Local recurrence after surgical resection of primary RPS is of most concern among the practicing surgeons in this specific surgical specialty which carries a high possibility of late death. Several factors have been claimed to be associated with local recurrence, i.e., grading of the sarcoma, histologic subtypes, tumor size, and adequacy of surgical resection. It is generally accepted that complete surgical resection (R0/R1) plays an important role in decreasing local recurrence [7, 9, 20, 21]. However, obtaining such complete resection is not an easy task in patients with RPS owing to its anatomical relationship to various important visceral and vascular structures. This is the reason why multivisceral resection has been added for en-bloc resection of the tumor to achieve adequate resection margins (R0/R1). We have adopted these surgical concepts since the beginning of the present study. Kidney, colon, and uterus with both fallopian tubes were the 3 most common organs resected (58%, 43%, and 21%, respectively). Our results with the 5-year overall survival rate of 37%, 5-year disease-free survival of 29%, and 5-year and 10-year recurrence rates of 71% and 95% have confirmed the infamous natural history of RPS of recurrence and eventually death despite a seemingly an appropriate treatment. We believe that all patients in this study received adequate and standard surgical procedures with 95% of R0/R1 resection and 87% of patients with >1 visceral organ resection. We realize that complete resection with possible multivisceral organs resection is an utmost important factor for the best outcome. Our relatively high 5-year and 10-year recurrence rate may be explained by: (1) The late presentation (patients came to our institution late with a median tumor size of 22 cm), (2) the majority of patients in our study (61%) had a high-grade tumor with 56% recurrence rate, and (3) unfavorable histologic subtypes of patients with liposarcoma (73%).

Bonvalot et al. [21] (the French group) and Gronchi et al. [22] (the Italian group) were among those who were credited as pioneers of introducing an aggressive surgical approach for RPS in 2009. Long-term results from both surgical groups were very encouraging [23, 24]. This extended procedure or “compartmental surgery” encompassed complete resection of tumor and normal surrounding tissue resulting in multivisceral resection which was more extensive than the standard procedure of removal of only organs obviously infiltrated by the tumor. Nevertheless, some controversies have been pointed out regarding the necessity and real benefits of routine employment of this aggressive surgery [25]. We understand that this extended procedure is at least based on the fundamental principle of surgical oncology, i.e., to achieve R0 resection which, as long as the surgical morbidity is acceptable, the procedure provides the best opportunity for cure. We have followed such a radical surgical approach with acceptable perioperative morbidity and mortality, with only a 16% postoperative complication rate and no 30-day post-operative mortality. Practically, when examining the surgical specimen removed from the abdomen after complete resection with multivisceral organs resection, no tumor itself was seen owing to its coverage with the resected normal tissue and resected visceral organs.

RPS is disreputable for its propensity to recur even though the original operation seems to be complete or adequate. This is why preoperative RT has been used in the majority of our patients since 2011 in order to reduce the local recurrence rate and possible improvement in survival as supported by many retrospective studies [26,27,28]. The survival benefit of preoperative RT is still debatable since a large randomized controlled trial (STRASS cohort) showed no survival benefit of preoperative RT, but subgroup analysis demonstrated better abdominal recurrence-free survival in low-grade liposarcoma [29, 30]. Currently, at our institution, we perform routine preoperative RT except in selected patients with a resectable well-differentiated liposarcoma, and in patients who need early surgery owing to the massive size of the primary RPS. In our experience with preoperative RT, the patients tolerated the RT well and subsequent radical surgery could be safely performed without any local complication of preoperative RT. Furthermore, the present study showed that preoperative RT was the only independent factor decreasing recurrence in the multivariate analysis. Hence, the use of preoperative RT may help reduce recurrence in selected patients. However, a future large trial is required before this recommendation can be made.

RPS is a rare disease, and treatment requires multi-departmental cooperation with excellent facilities which is not available in the majority of hospitals in Thailand. During 1.5 decades of studying patients with RPS, we observed some factors that may influence outcome and prognosis. Firstly, asymptomatic patients usually came to us late with a very large abdominal mass owing to the expandable capacity of the retroperitoneal space. This observation is a common presentation of patients with these rare tumors all over the world. Secondly, symptomatic patients also came to us late after multiple visits at other hospitals resulting in delayed diagnosis and treatment. This may be explained by the inadequate distribution of cancer centers in Thailand and the unfamiliarity of doctors for this rare disease leading to time-consuming unnecessary investigations and time-consuming interhospital referral. And thirdly, since we have increased our attention to treating patients with RPS, more patients with retroperitoneal mass were referred to us. Among them were patients with recurrence who received the first operation elsewhere. Almost all patients in this group underwent only retroperitoneal mass removal without visceral resection resulting in inadequate surgical margins. All these observations may reflect the need to initiate the foundation of sarcoma centers in Thailand. A specialized center with a high volume of patients would result in increasing the knowledge of RPS and improving the quality of care for patients with this rare and difficult-to-treat disease. We admitted that much more experience, knowledge, and organization of multidisciplinary teams are unquestionably needed to improve the standard of care of patients with RPS in Thailand.

The limitations of the present study were its retrospective design with a relatively small number of patients and a short follow-up time. Furthermore, 8 patients (21%) undergoing preoperative RT did not have a definitive diagnosis of the sarcoma subtype on the final pathological reports, hence, preoperative RT may have had some effects to the pathological examination of the resected specimens. However, to the best of our knowledge, this study is so far a pioneer one regarding primary RPS in Thailand which, we hope, will initiate a subsequent study of this rare malignancy in the future. Moreover, we believe that this study will increase the basic knowledge of patients with primary RPS in Asian Countries, of which, the number of publications is still low.

## Conclusions

We present our experience of treating 38 patients with primary RPS in Thailand. Our outcome in terms of local recurrence and survival was less favorable compared to other renowned international studies despite our aggressive surgical approach. Low-grade tumors had favorable prognosis and in liposarcoma, the subtypes of well-differentiated and myxoid had significantly lower recurrence rates than dedifferentiated, pleomorphic, and mixed type. Preoperative radiation may help reduce recurrence in selected primary RPS patients.
